# Genetic and functional characterization of HIV-1 Vif on APOBEC3G degradation: First report of emergence of B/C recombinants from North India

**DOI:** 10.1038/srep15438

**Published:** 2015-10-23

**Authors:** Larance Ronsard, Rameez Raja, Vaishali Panwar, Sanjesh Saini, Kumaravel Mohankumar, Subhashree Sridharan, Ramamoorthy Padmapriya, Suhnrita Chaudhuri, Vishnampettai G Ramachandran, Akhil C Banerjea

**Affiliations:** 1 Virology Laboratory, National Institute of Immunology, New Delhi, India; 2 Department of Microbiology, University College of Medical Sciences & Guru Teg Bahadur Hospital, Delhi, India; 3 Department of Virology, VP Chest Institute, University of Delhi, Delhi, India; 4 Department of Biochemistry and Molecular Biology, School of Life Sciences, Pondicherry University, Pondicherry, India; 5 Department of Pharmacology, Jawaharlal Institute of Postgraduate Medical Education and Research, Pondicherry, India; 6 Department of Human Physiology, University of Calcutta, Kolkata, India

## Abstract

HIV-1 is characterized by high genetic heterogeneity which is a challenge for developing therapeutics. Therefore, it is necessary to understand the extent of genetic variations that HIV is undergoing in North India. The objective of this study was to determine the role of genetic and functional role of Vif on APOBEC3G degradation. Vif is an accessory protein involved in counteracting APOBEC3/F proteins. Genetic analysis of Vif variants revealed that Vif C variants were closely related to South African Vif C whereas Vif B variants and Vif B/C showed distinct geographic locations. This is the first report to show the emergence of Vif B/C in our population. The functional domains, motifs and phosphorylation sites were well conserved. Vif C variants differed in APOBEC3G degradation from Vif B variants. Vif B/C revealed similar levels of APOBEC3G degradation to Vif C confirming the presence of genetic determinants in C-terminal region. High genetic diversity was observed in Vif variants which may cause the emergence of more complex and divergent strains. These results reveal the genetic determinants of Vif in mediating APOBEC3G degradation and highlight the genetic information for the development of anti-viral drugs against HIV. Importance: Vif is an accessory HIV-1 protein which plays significant role in the degradation of human DNA-editing factor APOBEC3G, thereby impeding the antiretroviral activity of APOBEC3G. It is known that certain natural polymorphisms in Vif could degrade APOBEC3G relatively higher rate, suggesting its role in HIV-1 pathogenesis. This is the first report from North India showcasing genetic variations and novel polymorphisms in Vif gene. Subtype C is prevalent in India, but for the first time we observed putative B/C recombinants with a little high ability to degrade APOBEC3G indicating adaptation and evolving nature of virus in our population. Indian Vif C variants were able to degrade APOBEC3G well in comparison to Vif B variants. These genetic changes were most likely selected during adaptation of HIV to our population. These results elucidate that the genetic determinants of Vif and highlights the potential targets for therapeutics.

Acquired immunodeficiency syndrome (AIDS) remains the most destructive pandemic disease among all infectious diseases. In India, first AIDS case was detected among sex workers^1^ and since it has spread to almost all the states of India. This rapid spread of infection is associated with high genetic heterogeneity of human immunodeficiency virus (HIV) due to high error rate of reverse transcription^2^, high titre virus production^3^, and fast replication kinetics involving high recombination rate^4^ which lead to emergence of highly divergent and circulating recombinant strains that permit virus to escape host immune responses and cause disease^5^.

HIV-1 virus is classified into groups, subtypes, sub-subtypes, circulating recombinant forms (CRFs) and unique recombinant forms (URFs). Major groups are M, N, O and the fourth group P was documented from a Cameroon woman living in Paris^6^. M group is the major group responsible for the AIDS pandemic which has been categorized into nine subtypes (A to D, F to H, J and K) and sub-subtypes (A1 and A2, F1 and F2)^7^. To date, as many as 72 CRFs and a growing number of URFs have been identified worldwide^8^ indicating the natural evolving tendency of virus.

The emergence of quasispecies and the enormous genetic diversity of HIV-1 virus have increased the burden of AIDS epidemic which is a major concern for developing countries like India. The current epidemiological reports based on global HIV-1 strains suggest that HIV-1 is rapidly evolving with the generation of large number of quasispecies^9^. During the course of natural infection, founder virus ultimately generates an extremely large pool of variant quasispecies within a single infected patient^10^. Previous studies focussing on HIV-1 epidemic in India showed predominance of subtype C followed by subtype B and some recombinants (A/C from Pune, C/ThaiB from Manipur and B/C from Karnataka)^11^ which were based on sequence analyses of env, gag and pol genes. It is very important to note that, there is no genetic information available for Vif gene which warrant for genetic study of Vif in our population.

One of the effective and promising ways to modulate HIV-1 infection is to target viral genes. Among various viral proteins, Vif is an accessory protein of size 23 kD which consists of functional domains namely F1 and F2 (APOBEC3F), G-box (APOBEC3G), FG-box (APOBEC3F and APOBEC3G), HCCH motif (zinc-binding domain), BC-box (Elongin BC complex), SOCS box (SOCS), and RNA motif (RNA-binding). Vif counteracts APOBEC3G^12^ and APOBEC3F^13^ proteins by recruiting cullin 5, elongin B, elongin C and the core binding factor beta (CBF-β) to form an E3-ubiquitin ligase complex leading to the degradation of APOBEC3G through ubiquitin proteosomal pathway^14^,^15^ resulting in the elimination of APOBEC3G from the host cells. In the presence of APOBEC3G, encapsidation of the virion fails to occur^16^. In the absence of Vif, APOBEC3G binds to HIV-1 and deaminates cytosine in the viral DNA negative sense strand, during reverse transcription leading to hypermutation and inactivation of provirus indicating the crucial role of Vif in maintaining the genetic integrity of virus^17^. It was reported that natural variation in Vif has differential impact on APOBEC3G/F degradation^18^ indicating the importance of functional characterization of Vif gene in our population.

The natural tendency of HIV-1 to undergo genetic variability contributes to its complexity which is a major challenge for developing therapeutics. HIV-1 genotypes have distinct geographical distribution, often with different clinical outcomes, prognosis and responses to Highly Active Antiretroviral Therapy (HAART)^19^. While the reasons for this remain a mystery, partial understanding of this issue is beginning to unfold. There are some evidences to suggest that emerging recombinants are associated with high viral loads and rapid HIV-1 spread^20^. Emergence of various recombinants during natural infection is due to the co-circulation of various subtypes within a single infected individual and also due to various *in-vivo* selection mechanisms^21^. Earlier reports represent the presence of B and C subtypes in Vpu and Vpr genes^22^,^23^ and predominance of subtype C in Env, Rev and Tat genes^24^,^25^ from North Indian population but there were no documented information available for Vif from North India and this is the first study to address this issue.

Here, we genetically characterized Vif gene from HIV-1 infected individuals of North India. This study revealed the co-circulation of Vif B and Vif C subtypes which might create a condition conducive for the generation of Vif B/C recombinants. The possible sequence similarity of our Vif variants with the global Vif variants were analysed by constructing phylogenetic tree. Various natural substitutions were observed which may have conferred high pathogenicity advantage to virus. Vif C variants resulted in little high APOBEC3G degradation in comparison to Vif B variants whereas Vif B/C recombinants were similar to Vif C in causing APOBEC3G degradation. This study discusses the influence of genetic variations of Vif on APOBEC3G degradation.

## Results

### Clinical data of HIV-1 infected patients

Blood of HIV-1 infected patients (n = 105) residing in Himachal Pradesh (n = 17), Punjab (n = 15), Haryana (n = 14), Chandigarh (n = 17), Uttar Pradesh (n = 20) and Delhi (n = 22) regions (North Indian regions) was collected during the period from 2004 to 2010. These subjects were from males (n = 40) and females (n = 34) with an average age of 30 years, eight mother to child infected pairs (n = 16), mothers with an average age of 33 years, children with an average of age 9 years. HIV-1 in 83% of subjects was due to heterosexual transmission and 17% of the subjects was due to vertical transmission from their mothers. The CD4 count for heterosexuals ranged from 62 to 1046 cells/mm^3^ (mean = 284 cells/mm^3^); for infected children it ranged from 152 to 589 cells/mm^3^ (mean = 514 cells/mm^3^) and for infected mothers it ranged from 183 to 811 cells/mm^3^ (mean = 252 cells/mm^3^). The clinical data of each individuals participated in this study is given [Table 1] and all HIV-1 infected individuals is shown [Supplemental file Table S1].

### Phylogeny based HIV-1 sub-typing and sequence similarity of Vif variants

**Figure 1:**
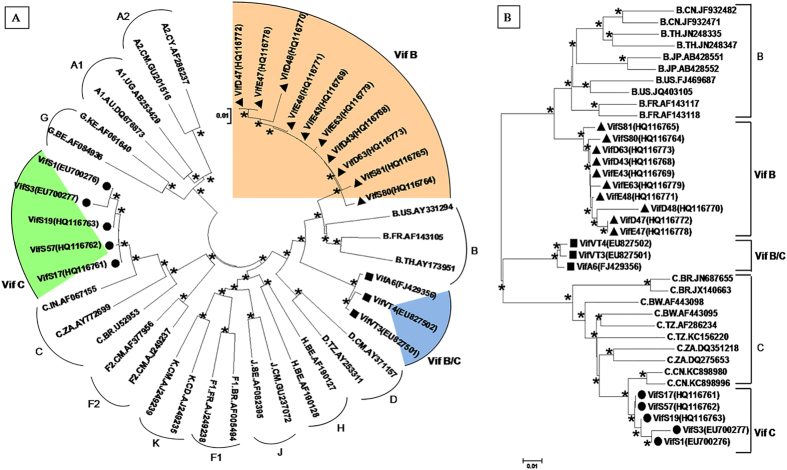
HIV-1 subtyping of Vif variants. (**A**) Phylogenetic tree of unique Vif variants with M (A to K including A1, A2, F1, and F2). (**B**) Phylogenetic tree of unique Vif variants with global subtype Vif B and Vif C reference sequences. Each reference sequence was labelled with subtype, followed by the country of isolation and accession number. Filled triangles represent B variants, filled circles represent C variants and filled rectangles represent B/C recombinants. The bootstrap probability (>60%, 1,000 replicates) was indicated with an asterisk (*) at the corresponding nodes of the tree and the scale bar represents the selection distance of 0.01 nucleotides per position in the sequence.

The amplicons of Vif from HIV-1 individuals were cloned and sequenced to determine the genetic variations. HIV-1 sub-tying was carried out to determine the genetic subtypes of Vif gene. Phylogenetic analysis revealed 50% of Vif variants clustered with Vif B, 35% of variants clustered with Vif C, and 15% of variants branched in between Vif B and Vif C indicating the presence of Vif B/C recombination, only representative unique variants of Vif B, Vif C and Vif B/C were used in the phylogenetic tree [Fig. 1A]. To identify the sequence similarity, phylogenetic tree was constructed by using maximum likelihood method with subtype Vif B from China (CN), France (FR), Japan (JP), Thailand (TH), United States (US) and subtype Vif C from China (CN), Botswana (BW), Tanzania (TZ), South Africa (ZA) and Brazil (BR). Vif C variants were closely related to subtype Vif C from China and South Africa; Vif B variants were closely related to subtype Vif B from France, America, Japan, Thailand and China indicating multiple introductions of Vif B strains from different geographic regions. This analysis indicates that Vif B/C recombinants are closely related to sequences from various populations which included France, Thailand, China and South African indicating the complexity of Vif B/C, only representative unique variants of Vif B, Vif C and Vif B/C were used in the phylogenetic tree [Fig. 1B]. Further, supporting phylogenetic tree was constructed with all 105 Vif variants shown [Supplemental file Figure S1A and S1B].

### Recombination events and nucleotide breakpoints in Vif variants

**Figure 2:**
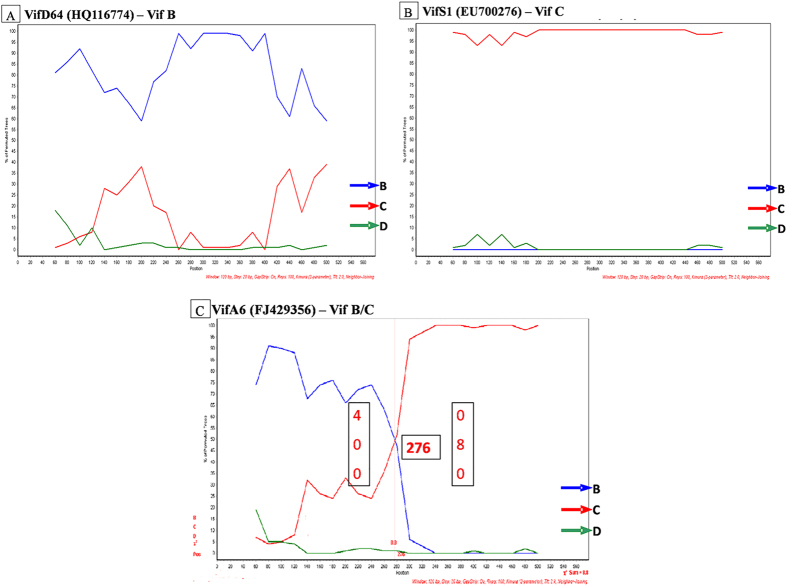
Bootscan analysis of Vif variants. (**A**) VifD64 is a representative of Vif B variant. (**B**) VifS1 is a representative of Vif C variant. (**C**) VifA6 is a representative of Vif B/C recombinant. Bootscan analyses were performed using consensus B, C and D shown in blue, red and green coloured arrows respectively with variants.

**Figure 3:**
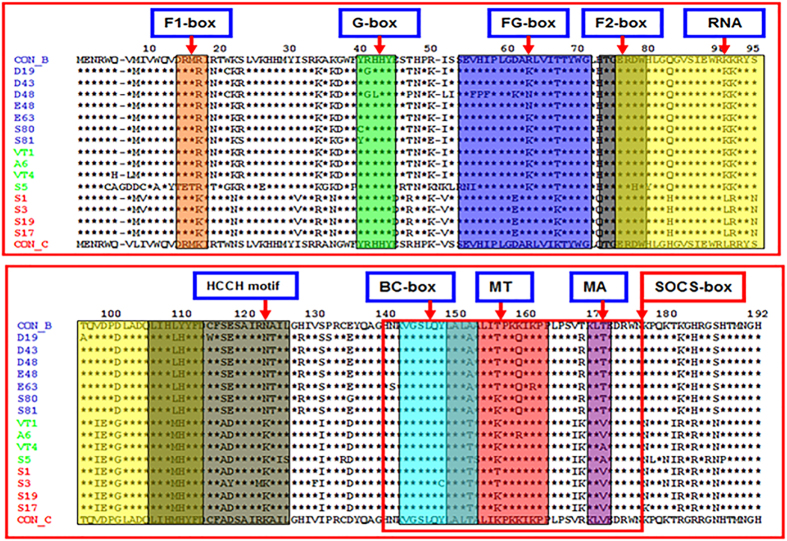
Multiple sequence alignment of Vif variants. Multiple sequence alignment for the predicted amino acid sequences of unique Vif variants with consensus Vif C and Vif B. In the alignment, amino acids identical to Vif C and Vif B are denoted by asterisk (*). Polymorphisms are denoted in alphabetical letters. Predicted major functional domains are represented at the top of the sequences within the boxes.

Bootscan analysis further confirmed that 50% of variants were closely related to subtype Vif B [Fig. 2A], 35% of variants showed relatedness with subtype Vif C [Fig. 2B]. It is noteworthy that 15% of variants were novel B/C putative recombinants in Vif gene (*p < 0.05) [Fig. 2C]. Informative site analysis of B/C recombinants showed that the N-terminal half consisted of 4, 0 and 0 values for Vif B, C and D subtypes respectively indicating closeness to Vif B and the C-terminal half consisted of 0, 16 and 0 values for Vif B, C and D subtypes respectively indicating closeness to Vif C. Further, sequences of all Vif variants were subjected to BLAST analysis in NCBI retroviruses genotyping tool^26^ using 300 p window size/40 bp step size which confirmed the previous subtyping results shown [Supplemental file Figure S2A, S2B and S2C]. This analysis was further verified using similarity plot in Recombination Identification Program (100 bp window size) and REGA HIV sub-typing tool. In addition, amino acid alignment of Vif variants with consensus Vif B and Vif C showed similarity with Vif B in Trp-rich region, F1-box, RNA binding region, G box, FG box and F2 box regions whereas HCCH box, BC box, SOCS box, MT and MA regions were identical to Vif C. The multiple sequence alignment of Vif variants indicated sequence diversity of Vif gene and revealed the presence of novel natural polymorphisms in North Indian population [Fig. 3].

### Natural polymorphisms in Vif variants

The nucleotide sequences of Vif were translated to amino acids, then aligned and compared with consensus Vif B and C. The reported Vif genetic determinants for biological activities were conserved in North Indian population; however various polymorphisms were observed at the functional domains of Vif which included fourteen high prevalent polymorphisms (R19N, S23R, G37D, H48N, R63K, V98I, D99E, A123T, H127R, K158Q, V166I, T167K, K176N and T180I) and eleven polymorphisms with less than 0.200 allele frequency were observed (Q6H, I9V, W21C, I31V, R41G, E45D, D61E, S95N, T96A, C114W and P131S) shown in [Table 2]. R19N change was observed in F1 box of about 76%; S23R, G37D, H48N, V98I and D99E changes were observed in RNA binding region of about 76%, 80%, 80%, 43% and 43% respectively; R63K change was observed in FG box of about 76%; HCCH region showed A123T and H127R in 53% of variants; K158Q was observed in multimerization region in 53% of variants; SOCS region showed V166I and T167K in 43% and 36% of variants respectively; K176N and T180I were observed in G box in 36% of variants.

The N-terminal tryptophan rich region displayed conservation at W5 and W11 and a polymorphism at W21C in 25% of variants. These highly conserved tryptophan residues are known for the efficient binding and degradation of APOBEC3G/F. W11 and W79 are critical for binding with APOBEC3F whereas W5, W21, W38 and W89 are important for Vif mediated APOBEC3G degradation^27^. These residues were highly conserved while R19N change was observed in 75% of variants. F1 box (D14RMR17) and G box (Y40RHHY44) are essential for binding and degradation of APOBEC3F and APOBEC3G respectively^28^. These regions were conserved but a change in R41G was found in 25% of variants. S23R, G37D and H48N were observed in 75% of variants which may modulate APOBEC3G/F degradation.

The N-terminal region has strongest affinity with RNA (RNA binding domain), forming a 40S messenger ribonucleic protein complex in infected cells. The reported data revealed that W11, Y30 and Y40 are crucial for RNA-APOBEC3G binding activity^29^ and these residues were conserved. Also, the region encompassing amino acids 63RLVITTYW70 and 86SIEWR89 is important for the formation of beta (β) strand which are essential for the normal expression of Vif leading to viral infectivity, and these amino acids were conserved in our population. The glutamic acid at 88^th^ position and the tryptophan at 89^th^ position were highly conserved which are known to enhance steady state expression of Vif in the host cells. FG-box is required for APOBEC3F and APOBEC3G interaction and was conserved except a polymorphism of R63K in 75% of variants. F2-box is essential for interaction with host APOBEC3F^30^ which were found to be conserved among North Indians.

Nuclear localization inhibitory signal (NLIS) from 90 to 93 amino acids (RKKR) was conserved in our Vif B variants. This region is important for importins binding, thus occluding nuclear import pathway and ensuring cytoplasmic localization of Vif whereas Vif C possessed RLRR sequences were highly conserved in our Vif C variants. This arginine rich region is involved in localization of Vif to the nucleus^31^. HCCH region with two conserved H/C pairs is predicted to form an alpha helix which binds Zinc ion. This region also binds Cullin5 selectively and therefore required for assembly of Vif–Cul5–E3 ubiquitin ligases to degrade APOBEC proteins^32^. Our Vif variants showed conservation at positions 108, 133 and 139 while C114W and H127R changes were observed in 12% and 50% of variants respectively. The hydrophobic residues at 115, 120, 123 and 124 positions are essential for interaction with Cullin5 which were conserved but a polymorphism was observed at 123^rd^ position with T in 50% of variants. It was reported that changes in these hydrophobic residues disrupt the binding of Zinc coordinating region to cullin5 leading to inhibition of APOBEC3G degradation^33^.

BC box (144SLQYLA149) is responsible for the binding with ElonginC to target APOBEC3G degradation. This region has been predicted to form an alpha helix that fitted into the hydrophobic pocket of ElonginC. Vif is known to bind ElonginC through BC-box and Cullin5 through hydrophobic residues within a zinc binding region formed by a conserved HCCH domain^34^; this region was conserved in our Vif variants. SOCS (suppressor of cytokine signalling proteins) box is essential for SOCS binding^35^ which led to inhibition of cytokine action. We observed K158Q, V166I, T167R/K and K176N changes in this region which might affect the viral infectivity. It was known that alanine substitution in 144SLQ149 region results in a loss of viral infectivity^30^. Multimerization region (161PPLP164) is known to play a vital role in oligomerization of Vif 36 involved in preventing APOBEC3G incorporation into virions^37^ and this region was conserved. The amino acid region (from 85 to 99 and 169 to192) is shown to mediate Vif-APOBEC3G binding; we observed S95N, T96A, V98I, D99E, and K176N changes in our population which might alter the viral infectivity.

**Figure 4:**
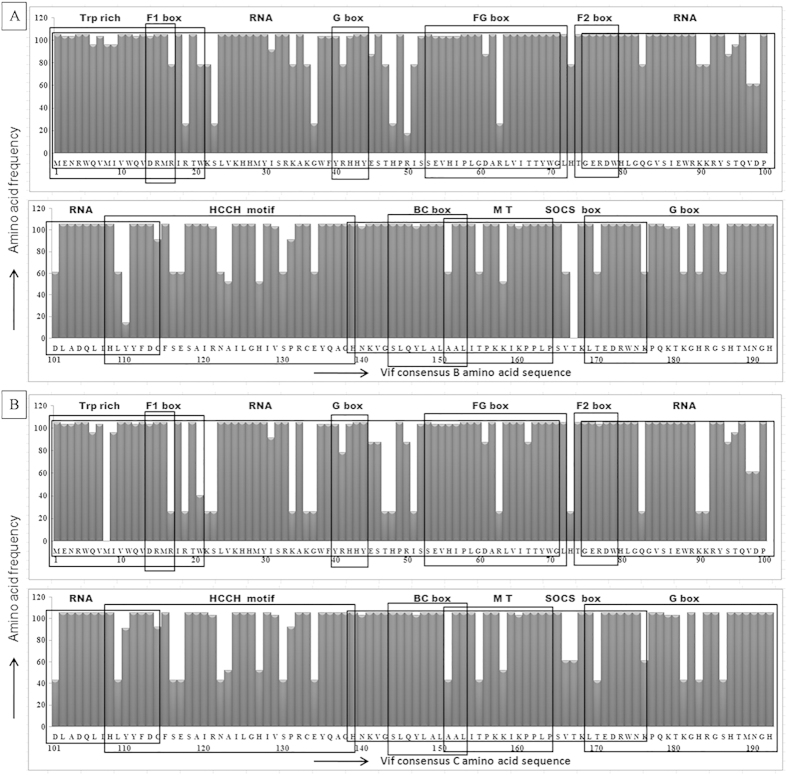
Amino acid sequence pattern of Vif variants. (**A**) Amino acid signature pattern of Vif variants with consensus Vif B. (**B**) Amino acid signature pattern of Vif variants with consensus Vif C. The X-axis represent the amino acid consensus sequence of Vif B and C with the functional domains viz. Trp-rich (N-terminal Tryptophan rich region from 1–21aa), F1-box (Interaction with host APOBEC3F from 14–17aa), RNA (RNA binding from 1–71aa and 75–114aa), G-box (Interaction with host from 40–44aa and 170–192aa), FG-box (Interaction with host APOBEC3F and from 54–72aa), F2-box (Interaction with host APOBEC3F from 74–79aa), HCCH motif (zinc-binding domain from 108–139aa), BC motif (Interaction with host elongin BC complex from 144–153aa), SOCS-box (Interaction with host SOCS from 139–176aa), MT (Multimerization from 151–164aa) and the Y-axis represent amino acid frequency observed in variants.

Despite numerous variations, alignment with consensus Vif B showed amino acid conservation at F1, F2, G, and FG boxes [Fig. 4A], notably R19N, S23R, G37D, H48N, R50K, R63K, Y110H and T167R polymorphisms were observed in North Indian population but not seen in Vif B consensus sequence. On the other hand, alignment with consensus Vif C showed amino acid conservation at F1, F2, G, and FG boxes whereas other regions showed certain changes [Fig. 4B]; sixteen amino acid polymorphisms were observed in Vif C variants that were not seen in Vif C consensus sequence. Our genetic sequence analysis of Vif variants revealed both synonymous and non-synonymous mutations with varying frequencies [shown in Table 2 and Supplemental Table S2]. Major non-synonymous mutations are shown in bold letters. We have observed high nucleotide conservation at the C-terminal of Vif than the N-terminus.

APOBEC3G is a DNA cytidine deaminase, which leads to dC→dU mutations in HIV-1 during Vif deficiency. Thus, creates a favorable environment for higher frequency of C→T mutations in Vif leading to manifestation in its functional activity. Therefore, it is interesting to investigate C→T mutations and its role in APOBEC3G degradation. In view of this, our genetic analyses revealed C→T mutations at four major nucleotide positions in the Vif gene viz. 138, 333, 391 and 539 positions. We observed more C→T mutations in Vif B than Vif C variants which could be one of the reasons for low APOBEC3G degradation ability of Vif B variants as compared to Vif C variants [Table 3].

### Phosphorylation and motif sites in Vif variants

**Figure 5:**
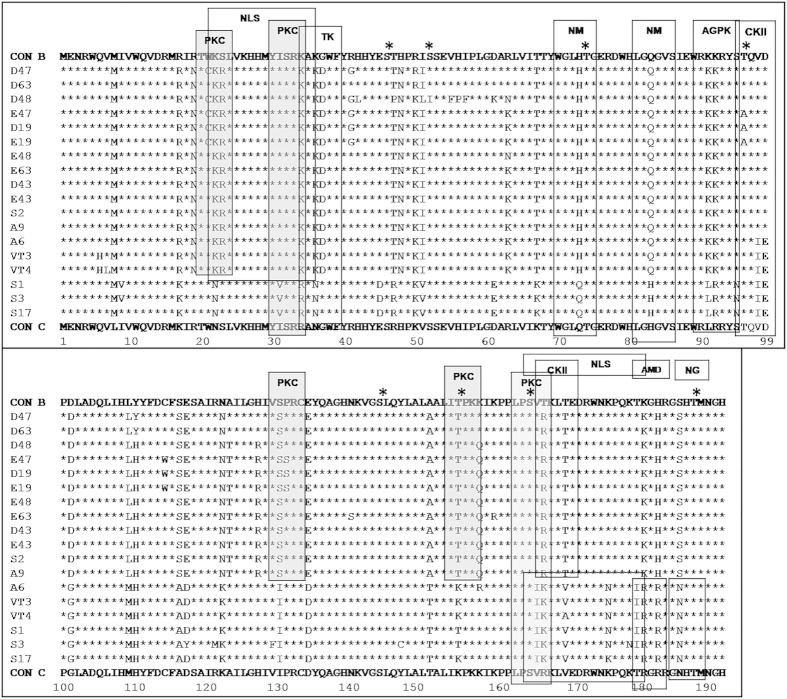
Motifs and phosphorylation sites in Vif variants. Vif variants show protein kinase C (PKC) sites (20–22aa, 32–34aa, 130–132aa, 155–157aa and 165–187aa), bipartite nuclear localization signal profile (NLS) sites (22–36aa and 167–184aa), tyrosine kinase (TK) site (33–40aa), N-myristoylation (NM) sites (71–76aa and 82–87aa), cAMP and cGMP dependent protein kinase (AGPK) sites (92–95aa and 167–170aa), casein kinase II (CKII) site (96–99aa), amidation (AMD) site (181–184aa) and N-glycosylation (NG) site (186–189aa). The phosphorylation sites (serine and threonine residues) were marked with asterisk (*) at the corresponding amino acid sites on the top of the consensus C sequence.

Vif is phosphorylated *in-vitro* and *in-vivo* by cellular kinases that are essential for viral replication^38^. The reported phosphorylation sites are T96, S144, T155, T170 and T188 and these sites were conserved in our variants. The disruption of T96 and S144 resulted in loss of Vif activity which is associated with restricted HIV-1 replication^38^. S144 and T188 were well conserved; T96 was conserved in 90% of variants while 10% of variants showed alanine (A) substitution. T155 was conserved in 86% of variants while 14% of variants showed lysine (K) change. T170 was conserved in 60% while 40% of variants showed valine (V) substitution. Phosphorylation site analysis using NetPhos2.0 server showed sites at S46, S52, S165, T74 and T96 which were conserved and shown with asterisk (*) at the corresponding amino acid sites [Fig. 5].

Motifs were identified using Motif scan showed conservation at amidation (AMD) and N-glycosylation (NG) motifs in Vif C and B/C variants but not in Vif B variants which was due to subtype B-specific genetic variations; cAMP and cGMP dependent protein kinase (cAGPK) and tyrosine kinase (TK) motifs were conserved in Vif B and B/C variants but not in Vif C variants which is due to subtype C-specific genetic variations. Notably, the important casein kinase II (CKII), N-myristoylation (NM) and protein kinase C (PKC) motifs were conserved in majority of variants irrespective of subtypes. Bipartite nuclear localization signal profile (NLS) motif is essential for nuclear import of viral proteins which were conserved in our population^39^ [Fig. 5].

### Synonymous versus non-synonymous changes in Vif variants

The dN/dS values for Vif variants were calculated using SNAP tool in HIV database which ranged in between 0.33 to 0.61 fold. All Vif variants including Vif B/C recombinants showed values less than one which is the indication of purifying selection. Further, to determine the synonymous versus non-synonymous changes that occurred between Vif B and Vif C variants, the average divergence of Vif B and C variants was generated for all Vif variants that also showed dN/dS values less than one which once again confirmed the purifying selection. This data states that Vif gene was not under positive selection among North Indians [Supplemental file Table S3], despite high genetic variations and B/C recombination.

### Expression of Vif proteins and APOBEC3G degradation by Vif variants

**Figure 6:**
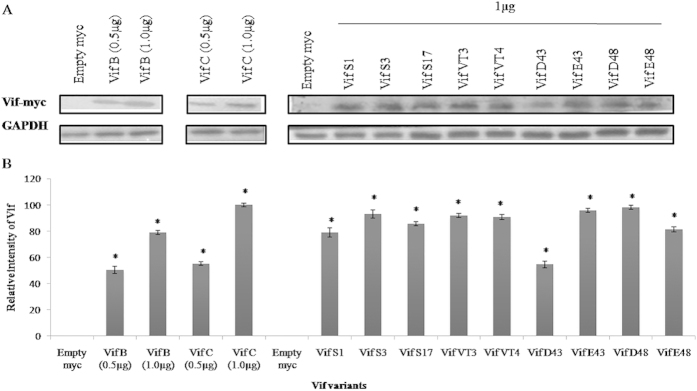
Protein expression of Vif variants. (**A**) Protein expression of Vif variants. (**B**) Relative intensity of Vif proteins. HEK 293T cells were transfected with Vif variants and empty pCMV-Myc vector used as a control. After 24 hr of transfection, cells were lysed and run on SDS-PAGE, and then expressed Vif proteins were detected by immunobloting with anti-Myc antibody. The relative protein intensity of Vif variants were measured by subtracting empty vector value. Densitometry represents the expression of Vif variants. The value of Vif C was set to 100% and the graph was made as relative values of Vif C expression. The error bar represents the standard deviation of Vif protein expression and the p-value ≤ 0.05 represents statistical significance of relative intensity of Vif protein expression as compared to empty myc (* denote significant difference).

**Figure 7:**
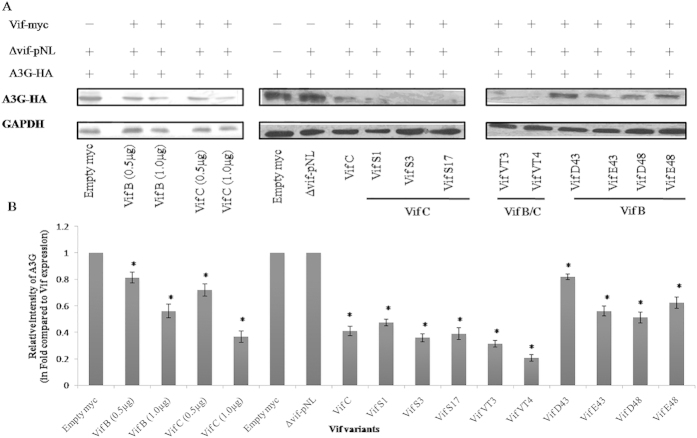
APOBEC3G degradation by Vif variants. (**A**) APOBEC3G protein expression induced by Vif variants. (**B**) Relative intensity of APOBEC3G. HEK 293T cells were co-transfected with Vif variants and APOBEC3G-HA. APOBEC3G-HA was used as positive control. After 24 hr of transfection, cells were lysed and run on SDS-PAGE, and then APOBEC3G protein was checked by immmunobloting with anti-HA antibody. The relative protein intensity of APOBEC3G degradation by Vif variants were measured by subtracting APOBEC3G-HA positive control. Densitometry represents the APOBEC3G degradation by Vif variants. The error bar represents the standard deviation of APOBEC3G degradation and the p-value < 0.05 represents statistical significance of relative intensity of APOBEC3G with the relative intensity of respective Vif (* denote significant difference).

Intracellular protein expression of Vif variants (VifS1, VifS3, VifS17, VT3, VT4, D43, E43, D48 and E48) were carried out in HEK 293T cells by western blotting. It was observed that all Vif variants were expressed well (**p < 0.05) [Fig. 6 and also Supplemental file Figure S3]. This protein expression were independent from the genetic variations occurred within the functional domains of Vif. The ability of these variants to degrade APOBEC3G was determined by measuring the relative intensity of APOBEC3G. This was achieved by co-transfecting of pCMV-Myc-Vif variants and APOBEC3G-HA in HEK 293T cells. The APOBEC3G-HA alone was used as positive control to determine the potentiality of APOBEC3G degradation by Vif variants. The relative intensities of A3G were normalised with the relative intensities of respective Vif myc variants and the values were represented as ‘relative intensity of A3G in folds compared to Vif expression’. Comparatively, Vif C variants showed quite high level of APOBEC3G degradation (*p < 0.05) than Vif B variants. Perhaps, this differential APOBEC3G degradation induced by Vif variants might be due to the genetic variations in between subtype specific variants. Vif B/C recombinants resulted in similar level of APOBEC3G degradation in comparison to Vif C but a little higher (*p < 0.05) than Vif B variants [Fig. 7 and also Supplemental file Figure S4] which could be due the presence of C-terminal region (from subtype Vif C). It was already acknowledged fact that C-terminal of Vif plays a vital role in APOBEC3G degradation^40^,^41^. Six to eight variants from each representative group (Vif C, Vif B, Vif B/C) were also checked for intracellular protein expression and APOBEC3G degradation; it was observed that similar levels of expression and degradation were observed.

## Discussion

This study shows the genetic architecture of HIV-1 Vif comprised of B and C subtypes. This co-circulation of subtypes within a specific population led to the emergence of novel B/C recombinants in the ORF of Vif among North Indians which was observed to be statistically significant (**p < 0.05) i.e, 15% recombinants were observed. Thus, the proportion of recombinants have increased marginally (~3 to 5%) in the last few years implying the stable emergences of recombinants indicating the evolving tendency of the virus in our country. Viral genetic recombination analysis also confirmed the existence of B/C recombinants with precise breakpoint in Vif gene. These recombinants may contribute for generation of more complex viruses in our population; therefore it is essential to understand the genetic makeup of circulating recombinants and their prevalence in North India which will help in designing effective therapeutics against various circulating strains. Phylogenetic analysis of Vif C variants showed sequence similarity with South African C variants whereas Vif B variants showed sequence similarity with the subtype B from different geographic regions that have resulted from multiple introductions of HIV-1 strains from China and other countries viz. Thailand, France and America, probably due to itinerant travellers from different countries. Our data elucidates the importance of phylogenetic and recombination analyses in determining the sequence similarity between our variants with the highly prevalent global strains of HIV-1 and contribute to the better understanding of virus with respect to natural selection and adaption of viral strains.

Polymorphism analysis showed certain conserved substitutions which may favour virus for its optimal activities in causing pathogenicity in host cells. Notably, these substitutions may involve in down-regulating or enhancing the functional activities of Vif and might also aid in the generation of more complex and divergent strains. Therefore, targeting the natural substitutions using RNAi mechanism may help in modulating HIV-1 infection. It is important to note that this study is an attempt to analyze Vif variants from ART naïve and ART receiving patients of North India, in relation to the contribution of this trait to pathogenicity, viral evolution and possible implications for ART regimen and vaccine design. Interestingly, low genetic diversity and lesser recombinants were observed among HIV-1 infected individuals receiving ART who showed improved CD4 counts and decreased viral loads implying the importance of ART in restoring immunity.

Domain conservation analysis based on sequence profiles of 105 HIV-1 infected patients from North India revealed the conservation at various functional domains viz. APOBEC3G/F, Cullin5, ElonginC and SOCS binding domains highlighting the target sites for designing Anti-viral drugs. Motif sites analysis showed conservation at various motifs in variants; remarkably identified nine phosphorylation sites were conserved. The dN/dS analysis suggests purifying selection implying genetic integrity of Vif to maintain the functional activity of Vif. It is necessary to have a matrix of variants in HIV-1 affected population since it will have a predictive value in determining the type of Anti-viral drug targets. Based on sequence analysis of Vif variants in a specific population, it is possible to map cytotoxic T lymphocyte (CTL) epitopes and enhance immune response of HIV-1 infected individuals against virus.

Subtype-specific differences in biological activity of viruses are an acknowledged aspect of HIV-1 pathogenesis^42^; inter-subtype recombinants can influence AIDS progression rate but the viral or cellular components responsible for these differences are not clearly defined. Vif is known to display subtype-specific differential activity during natural HIV-1 infection^18^; this study underscores the importance of genetic and functional characterization of Vif gene which may help in a better understanding of evolving properties of HIV-1 virus. Vif C variants (Vif S1, S3 and S17) resulted in a little high APOBEC3G degradation as compared to Vif B variants which could be due to the genetic variation at the C-terminal of Vif and this region was reported for several post-translational modifications leading to APOBEC3G degradation^43^.

Vif targets APOBEC3G and degrades it through ubiquitination pathway while APOBEC family proteins play a central role in intrinsic host cellular defences against HIV infection^14^. To explore the relationship between Vif-mediated APOBEC3G degradation, dual transfection with Vif variants and APOBEC3G was carried out in HEK 293T cells. This assay clearly showed that Vif B variants (Vif D43, E43, D48 and E48) degrades APOBEC3G whereas Vif B/C recombinants (VT3 and VT4) carrying the N terminal region of Vif B and the C terminal of Vif C resulted in a little high APOBEC3G degradation as compared to Vif B and similar levels to Vif C confirming the vital role of C-terminal region in inducing APOBEC3G degradation. This differential potential of Vif variants was due to the genetic variations in Vif B, Vif C and Vif B/C variants. This study on Vif from HIV-1 infected individuals (n = 105) revealed Indian Vif sequences (VifS1, VifS3, VifS17, VT3, VT4, D43, E43, D48 and E48) which were highly conserved. These matrixes of variants can be used in a combination for the understanding of Vif-host relationship and to develop targets against HIV in our population.

This study deciphers that HIV-1 virus continue to evolve in the local population, but current pace of evolution is not so alarming, perhaps due to ART intervention. Whether the dynamics of variation in the evolving population of viruses will retain its current pace or will it be altered is difficult to predict. Whether host immune response is adequate to contain the variations in the evolving virus and whether other factors (host immune response, environment, etc) are likely to influence it remains to be seen. Coinfections such as Tuberculosis may have profound impact on the host immune response thereby compelling the virus to evolve new strategies to synergize with Mycobacterium tuberculosis. This could imply an incessant alteration, in the genetic and functional characteristics of the virus which may be subtle or gross for finding a better-fit to the situation in the host encountered by the virus. Thus, periodic analysis of the virus for selection pressure-dictated variants becomes necessary to determine the trend of viral evolution, if the variants are likely to pose a public health problem, if changes are necessary in the modality of ART, if newer viral targets can be obtained for prophylactic or therapeutic intervention and finally, if the biology of the virus is underpinning a new element in basic sciences. Our data indicates the genetic and functional role of Vif in causing APOBEC3G degradation, targeting Vif by utilizing novel RNAi technology will help in perturbing the viral infectivity. This study revealed various natural genetic determinants of Vif in mediating the functional activity of Vif in our population, thus this study will immensely help in designing the potential targets based on Vif-APOBEC3G relationship against HIV/AIDS. Taken together, our data demonstrates that HIV-1 virus is evolving relevant to adaptation among North Indians through its ability to generate advantageous substitutions and recombinants in Vif gene to enhance its specific functional activities in the host cells.

## Materials and Methods

### Patient selection and ethics statement

HIV-1 infected patients (n = 105) were collected from North India who were registered and monitored at the immunodeficiency clinics of Guru Teg Bahadur (GTB) hospital, Delhi and Post Graduate Institute of Medical Education and Research (PGIMER), Chandigarh during the period from 2004 to 2010. Number of males (45%) and females (38%) were chosen for this study along with vertical transmission variants (17%). This study was approved by Research Project Advisory Committee, Institutional Biosafety Committee and Institutional Ethical Committee from Human research of University College of Medical Sciences and Guru Teg Bahadur Hospital, Delhi, India and from Post Graduate Institute of Medical Education and Research, Chandigarh, India. These institutes are mentored by National AIDS Control Organization (NACO), Ministry of Health and Family Welfare, Government of India that provides free of cost ART to HIV-1 seropositive patients under a structured HIV/AIDS Control Programme. These ethics committees approved the written informed consent which obtained from HIV-1 infected patients and from guardians of HIV-1 infected children participants involved in this study. The methods were carried out in “accordance” with the approved guidelines.

### RNA isolation, Reverse Transcription-PCR and PCR

Total RNA was extracted from peripheral blood mononuclear cells (PBMCs) of HIV-1 infected individuals using Trizol reagent (Invitrogen). PBMCs were washed with PBS and 1 ml of Trizol was added to 50–100 mg cells. After incubation for 5 minutes at 37 °C, 200 μl of chloroform was added and mixed by vortexing. This was incubated for 5 minutes at 37 °C and centrifuged at 13,200 rpm for 15 minutes. The upper aqueous phase was transferred to the fresh tube and 500 μl of isopropanol was added to precipitate RNA. The mix was incubated at 37 °C for 10 minutes and centrifuged at 13,200 rpm for 10 minutes at 4 °C. The supernatant was discarded and pellet was washed with 75% ethanol. The pellet was air dried and dissolved in nuclease free water by heating at 55°–60 °C for 10 minutes. This RNA was reverse transcribed to form complementary DNA (cDNA) using ImProm-II^TM^ Transcription system (Promega). 1 μg of RNA was reverse transcribed into cDNA using 20 pmol/μl of random primers and incubated at 70 °C for 15 minutes and kept at 4 °C. Reverse transcription mix containing 1X reaction buffer, MgCl_2_, dNTPs, rRNasin RNase inhibitor and reverse transcriptase was added to it and incubated at 25 °C for 5 minutes, 42 °C for 1 hour, 70 °C for 15 minutes and kept at 4 °C. 5 μl of cDNA product was used for amplification of Vif using the following primers:

Forward primer: 5′-GCGGATCCATGGAAAACAGATGGCAGG-3′

Reverse primer: 5′-GCCTCGAGCTAGTGTCCATTCATTGTATGG-3′

PCR was carried out in a 15  μl reaction volume. The reaction mixture contained 500 ng genomic DNA (2.0 μl), 10X PCR Buffer (1.5 μl), 10 mM dNTP mix (0.37 μl), 1  μl of each primer (25 pmol), 0.25  μl of Takara Taq DNA polymerase and 8.88 μl of DNase/RNase free water. PCR conditions for the above primer sets were as follow: Initial denaturation at 94 °C for 5 minutes (1 cycle), 30 cycles of denaturation at 94 °C for 30 seconds, annealing at 65 °C for 45 seconds and extension at 72 °C for 40 seconds, and a final extension at 72 °C for 5 minutes (1 cycle). PCR amplified products were analyzed on 1.5% agarose gel.

### Cloning and sequencing

The gel purified PCR products were cloned in pGEM-T Easy vector system. The ligation reaction was incubated at 4 °C for 10 hours and the ligation mix was then plated on LB ampicillin plates with E.coli DH5α strain as host. The plates were then incubated overnight at 37 °C. The positive clones were selected by picking a single colony and grown in 5 ml LB Broth with ampicillin antibiotic (100 μg/ml) and incubated overnight at 37 °C. Plasmid DNA was isolated from the culture by QIAprep Spin Mini Kit. The positive clones were screened by restriction digestion of plasmid DNA with EcoRI in a 10 μl reaction volume at 37 °C for 2 hours. The digested products were analyzed on 1.5% agarose gel after electrophoresis and the amplified bands were screened for positive clones after restriction digestion. Five positive clones from each individual were sequenced from LabIndia and SciGenom laboratories by dideoxy chain termination method. The cloning and sequencing were carried out twice for the positive clones to avoid PCR generated errors. A known HIV-1 NL4-3 sequence was included for PCR amplification as a control to assess errors generated by Takara Taq polymerase.

### HIV-1 sub-tying and sequence similarity analysis

The nucleotide sequences were assembled and checked for errors by using BLAST program (NCBI, USA) to search for sequence similarities to previously reported sequences in the databases and to eliminate potential laboratory errors. These sequences were aligned with consensus sequences of HIV-1 strains of all subtypes using the ClustalW 2.1^44^. The phylogenetic analyses were performed using neighbour joining method (sub-typing) and maximum likelihood method (sequence similarity)^45^ with Kimura two parameter distance matrix in MEGA5 software with M group subtypes (A-K) and sub-subtypes (A1, A2, F1 and F2). The reliability of node was tested using the bootstrap method with 1000 replicates. The phylogenetic trees were constructed for Vif representative variants (12 Vif B variants, 3 Vif C variants and 3 Vif B/C recombinants) from North India and reference sequences which were retrieved from HIV Sequence database that includes subtype B and C sequences obtained from different parts of the world include America, Japan, France, China, Thailand, Brazil, Kenya, Botswana, Zambia, South Africa and other countries. The basic criterion for choosing these reference sequences is that to cover all regions in worldwide for analysis in order to determine the sequence similarity with global reference sequences.

### Recombination events analysis and dN/dS ratio calculation

The presence of possible recombination events were confirmed by bootscan analyses of Vif B, C and D consensus sequences using 120 bp window size/20 bp step size with kimura two parameter in Simplot version 3.5.1 to predict the breakpoints within variants. Further, sub-typing and recombination events were verified by using genotype tool for retroviruses in the NCBI and the results were confirmed by using REGA HIV-1 sub-typing tool version 2.0 and Recombinant Identification Program (RIP) in the HIV databases. The type of selection pressure that might have occurred in Vif variants was calculated by the average dN/dS values within the predicted subtypes using SNAP v1.1.0 (Synonymous Non-synonymous Analysis Program). SNAP calculates non-synonymous (dN) and synonymous (dS) substitution rates based on a set of codon aligned nucleotide sequences (dN/dS)^46^.

### Polymorphisms identification and amino acid conservation analysis

The nucleotide sequences of Vif were translated into amino acid sequences by GeneRunner. The multiple sequence alignments were made for all variants with consensus B and C using ClustalW 2.1. It is already well known that subtype C is highly prevalent in India followed by subtype B and choosing consensus B and C sequences for analysis purpose is the idea behind the subtype prevalence in India and were retrieved from HIV Sequence database. Novel polymorphisms were identified and their allele frequencies were calculated from total variants (n = 105). The amino acid sequence conservation was determined from the multiple sequence alignments of variants with the corresponding consensus B and C sequences using sequence alignment tool in MEGA5 software.

### Motifs and phosphorylation sites analyses

The motifs and phosphorylation sites in variants were analyzed using Motif scan and NetPhos2.0 Server by comparing consensus B and C sequences^25^.

### Cell culture, transfection, plasmids and antibodies

HEK-293T (Human Embryonic Kidney 293 cells; NIH AIDS Reagent Programme) were maintained in Dulbecco’s modified Eagle’s medium (DMEM) with 10% fetal bovine serum and 100 units penicillin, 0.1 mg streptomycin and 0.25 μg amphotericin B per ml at 37 °C in the presence of 5% CO_2_. All transfections were performed using Lipofectamine 2000 (Invitrogen) reagent. pCMV-Myc-Vif, pCMV-HA-Ub, pCMV-Myc-A3G were used in the experiments. Anti-Myc monoclonal antibody (Clontech), anti- HA tag polyclonal antibody (Clontech), anti-GAPDH antibody (Cell Signalling Technology), anti-vif monoclonal antibody (NIH AIDS Reagent Programme), anti-Rabbit IgG conjugated to HRP (Jackson Immunoresearch), anti-Mouse IgG conjugated to HRP (Jackson Immunoresearch) were used in the experiments.

### Immuno-blotting

HEK-293T cells were co-transfected with 1 μg of pCMV-myc expressional vector encoding Vif variants including three Vif C variants (VifS1, VifS3 and VifS17), two Vif B variants (VT3 and VT4) and four Vif B/C recombinants (D43, E43, D48 and E48), and 1 μg of pCMV-HA-A3G. pCMV-HA-A3G alone was used as control. After 36 hours of co-transfection, cells were harvested and lysed in RIPA lysis buffer (1% NP-40, 20 mM TrisCl, pH 7.5, 150 mM NaCl, 1 mM Na2EDTA, 1 mM EGTA, 1% Sodium deoxycholate, 1 mM Na3VO4). Protein concentration was measured using BCA protein assay kit (Pierce, Thermo Scientific). Equal amount of proteins were resolved by 10% SDS PAGE (Sodium Dodecyl Sulfate- Poly-Acrylamide Gel Electrophoresis) and were transferred to nitrocellulose membrane. The membranes were blocked with 1% BSA (Bovine Serum Albumin; sigma) and 5% non fat dry milk (Himedia Laboratories) in 1X PBS (Phosphate Buffer Saline) and washed thrice with 1X PBS containing 1% Tween 20 (PBST; MERCK). Then the membranes were probed with primary antibody (Anti-Myc antibody, Anti-Vif antibody and Anti-HA antibody) washed with PBST and probed with horseradish peroxide (HRP)-conjugated secondary antibody (Anti-mouse antibody). Blots were developed using ECL (Enhanced Chemi-luminiscent) reagent. GAPDH was used as a loading control in all cases (as described^47^,^48^,^49^).

### Statistical analysis

All our data were analyzed using the SPSS 7.5-Windows Students version software (SPSS Inc., Chicago, IL, USA). One-way ANOVA followed by Fisher’s Least Significant Difference (LSD) test was used to determine the statistical significance between groups for all the measurements. The level of p < 0.05 value was considered to be statistically significant (as described^48^,^49^,^50^).

### Accession Numbers for Unique Vif variants

[GenBank: HQ116761-HQ116779, HQ116776-HQ116777, EU827499-EU827502, EU700276-EU700278, EU659813-EU659814, FJ429356]

## Additional Information

**How to cite this article**: Ronsard, L. *et al.* Genetic and functional characterization of HIV-1 Vif on APOBEC3G degradation: First report of emergence of B/C recombinants from North India. *Sci. Rep.* **5**, 15438; doi: 10.1038/srep15438 (2015).

## Supplementary Material

Supplementary Information

## Figures and Tables

**Table 1:** Clinical data of HIV-1 infected patients used in Vif study.

**Data**	**Mean**	**Range**	**Percentage**
**Age (yrs)**	30	4 to 50	—
**Sex (%)**	—	—	45% men, 38% women, 17% mother to child pairs
**Route of transmission (%)**	—	—	83% heterosexual and 17% vertical transmission
**Positive since detection (yrs)**	6	2004 to 2010	—
**ART status (%)**	—	—	ART recipients (43%) and ART naive (57%)
**CD4 counts (cells/mm**^3^)	284 cells/mm^3^ for heterosexuals, 514 cells/mm^3^ for infected children and 252 cells/mm^3^ for infected mothers	62–1046 cells/mm^3^ for heterosexuals, 152–589 cells/mm^3^ for infected children and 183–811 cells/mm^3^ for infected mothers	—

**Table 2:** Polymorphisms in Vif variants from North India.

**SL.NO**	**Nucleotide change**	**Amino acid change**	**Functional domains**	**Allele frequencies**
1	G18T	Q6H	Tryptophan rich	0.111
2	A25G	I9V	Tryptophan rich	0.111
3	A57C	R19N	F1 box	0.722
4	G63C	W21C	F1 box	0.166
5	T69A	S23R	RNA region	0.666
6	A91G	I31V	RNA region	0.277
7	A111C	G37D	RNA region	0.722
8	A121G	R41G	G box	0.166
9	A135C	E45D	G box	0.277
10	C142A	H48N	RNA region	0.722
11	T183G	D61E	RNA region	0.333
12	G188A	R63K	FG box	0.722
13	G284A	S95N	RNA region	0.277
14	A286G	T96A	RNA region	0.111
15	G292A	V98I	RNA region	0.444
16	C297A	D99E	RNA region	0.444
17	T342G	C114W	HCCH	0.111
18	G367A	A123T	HCCH	0.555
19	A380G	H127R	HCCH	0.555
20	C391T	P131S	HCCH	0.111
21	A472C	K158Q	MT region	0.555
22	G496A	V166I	SOCS	0.444
23	G500A	T167K	SOCS	0.444
24	G528T	K176N	G box	0.333
25	C539T	T180I	G box	0.333

**Table 3:** C→T mutations in Vif variants from North India.

**Mutations**	**Vif C (S1, S3 and S17)**	**Vif B/C (VT3 and VT4)**	**Vif B (D43, E43, D48 and E48)**
C138T	✘	✓	✓
C333T	✘	✘	✓
C391T	✘	✘	✓
C539T	✓	✓	✘
A3G degradation	High	High	Low
