# Supplementary material for: Genetic and functional characterization of HIV-1 Vif on APOBEC3G degradation: First report of emergence of B/C recombinants from North India
Source: Sci Rep. 2015 Oct 23;5:15438. doi: 10.1038/srep15438 (PMC4616021; doi:10.1038/srep15438)
Supplement: Supplementary Information [file srep15438-s1.pdf]

Supplemental files:

Genetic and functional characterization of HIV-1 Vif on APOBEC3G degradation: First report of emergence of B/C recombinants from North India

Larance Ronsard*[1,2,#], Rameez Raja[1], Vaishali Panwar[2], Sanjesh Saini[3], Kumaravel Mohankumar[4], Subhashree Sridharan[4], Ramamoorthy Padmapriya[5], Suhnrita Chaudhuri[6], Vishnampettai G Ramachandran[2] and Akhil C Banerjea[1*]

Authors' affiliation

[1]Virology Laboratory, National Institute of Immunology, New Delhi, India,

[2]Department of Microbiology, University College of Medical Sciences & Guru Teg Bahadur Hospital, Delhi, India,

[3]Department of Virology, VP Chest Institute, University of Delhi, Delhi, India.

[4]Department of Biochemistry and Molecular Biology, School of Life Sciences, Pondicherry University, Pondicherry, India.

[5]Department of Pharmacology, Jawaharlal Institute of Postgraduate Medical Education and Research, Pondicherry, India

[6]Department of Human Physiology, University of Calcutta, Kolkata, India

# Current affiliation: Division of Infectious Diseases, Boston Children's Hospital, Harvard Medical School, Boston, MA, USA.

***Correspondence authors**

Dr. Akhil C. Banerjea, Chief Staff-Scientist VII, Laboratory of Virology, National Institute of Immunology, Aruna Asaf Ali Marg, New Delhi-110067, India; Tel No: +91-011-26703616; Fax No: +91-011-26742125; Email IDs: akhil@nii.res.in, akhil@nii.ac.in.

Dr. Larance Ronsard, Post Doctoral Research Fellow, Division of Infectious Diseases, Boston Children's Hospital, Harvard Medical School, 300 Longwood Avenue, Boston, MA 02115, USA. Mobile No: +1-857-770-8126; Email IDs: laraphds@gmail.com & laranceronsard@gmail.com

Running Title: Functional implications of Indian Vif variants.

A

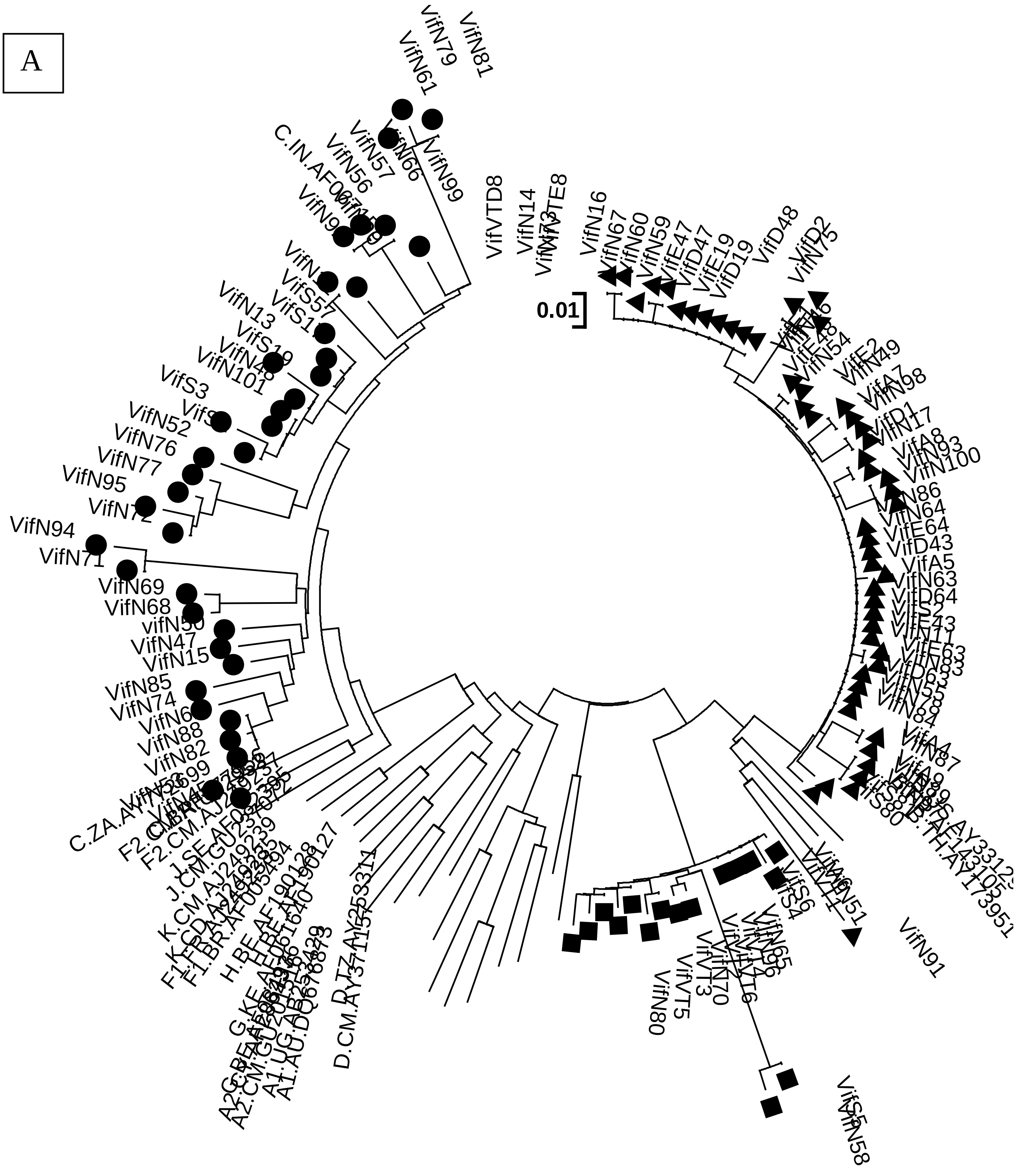

0.01
VifN81
VifN79
VifN61
VifN99
VifVTD8
VifN14
VifTE8
VifN16
VifN67
VifN60
VifN59
VifE47
VifD47
VifE19
VifD19
VifD48
VifD2
VifN75
VifN54
VifE2
VifN49
VifA7
VifN98
VifD1
VifN17
VifA8
VifN93
VifN100
VifN86
VifN64
VifE64
VifD43
VifA5
VifN63
VifS2
VifN91
VifS5
VifN58
VifS6
VifS4
VifN51
VifN65
VifN70
VifVT3
VifVT5
VifN80
D.CM.AY371157
C.ZA.AY772699
F2.CM.AJ249237
J.SE.AF082394
H.BE.AF190128
A1.UG.AB253429
A1.AU.DQ676873
VifN94
VifN71
VifN69
VifN68
vifN50
VifN47
VifN15
VifN85
VifN74
VifN88
VifN82
VifN95
VifN72
VifN77
VifN76
VifN52
VifS3
VifN101
VifN13
VifS19
VifN56
VifN57

B

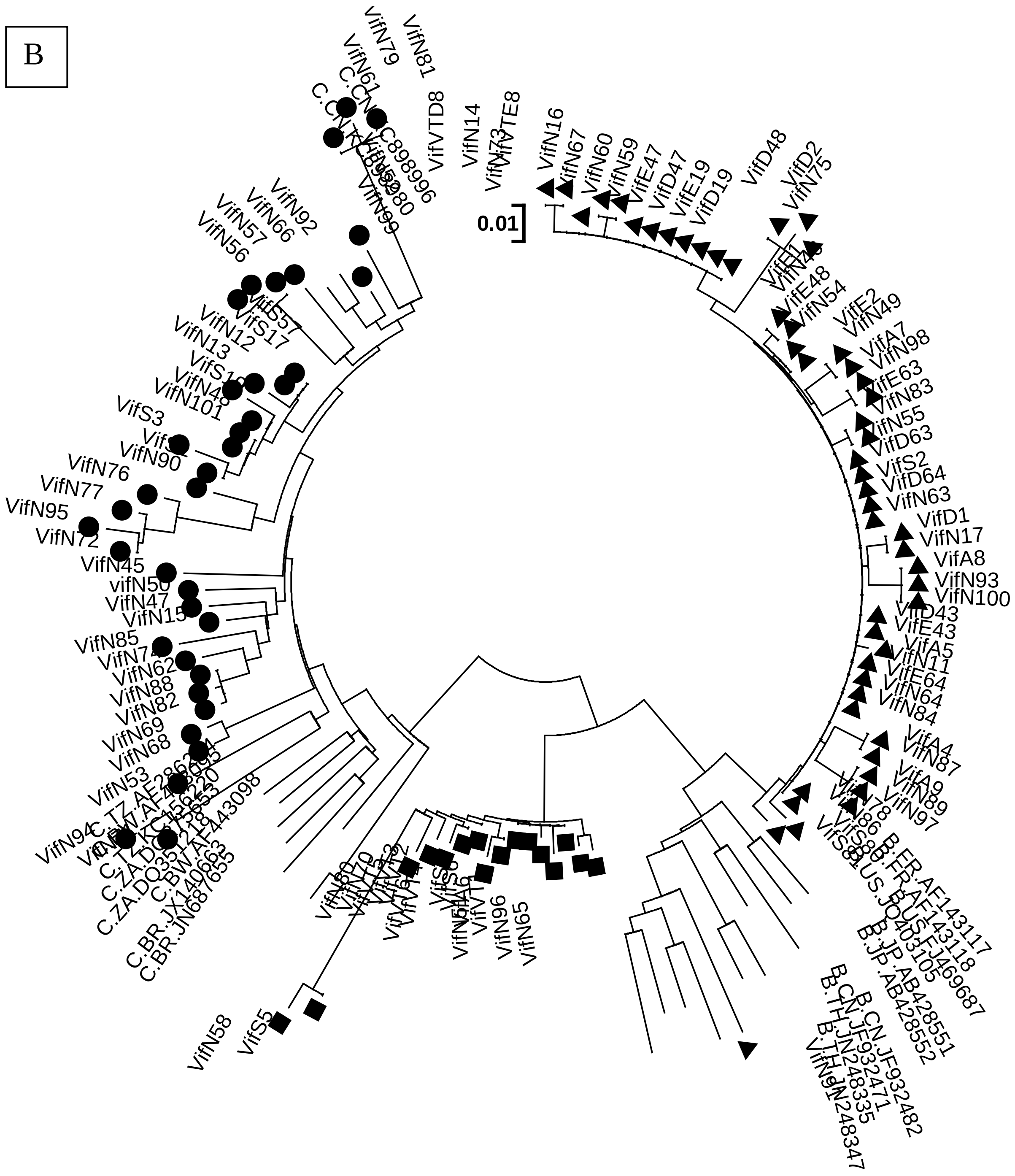

Supplemental Figure S1. **HIV-1 subtyping of Vif variants.** A) Phylogenetic tree of 105 Vif variants with M (A to K including A1, A2, F1, and F2). B) Phylogenetic tree of 105 Vif variants with global subtype Vif B and Vif C reference sequences. Each reference sequence was labelled with subtype, followed by the country of isolation and accession number. Filled triangles represent B variants, filled circles represent C variants and filled rectangles represent B/C recombinants. The bootstrap probability (>60%, 1,000 replicates) was indicated with an asterisk (*) at the corresponding nodes of the tree and the scale bar represents the selection distance of 0.01 nucleotides per position in the sequence.

A **VifD64 (HQ116774) – Vif B**

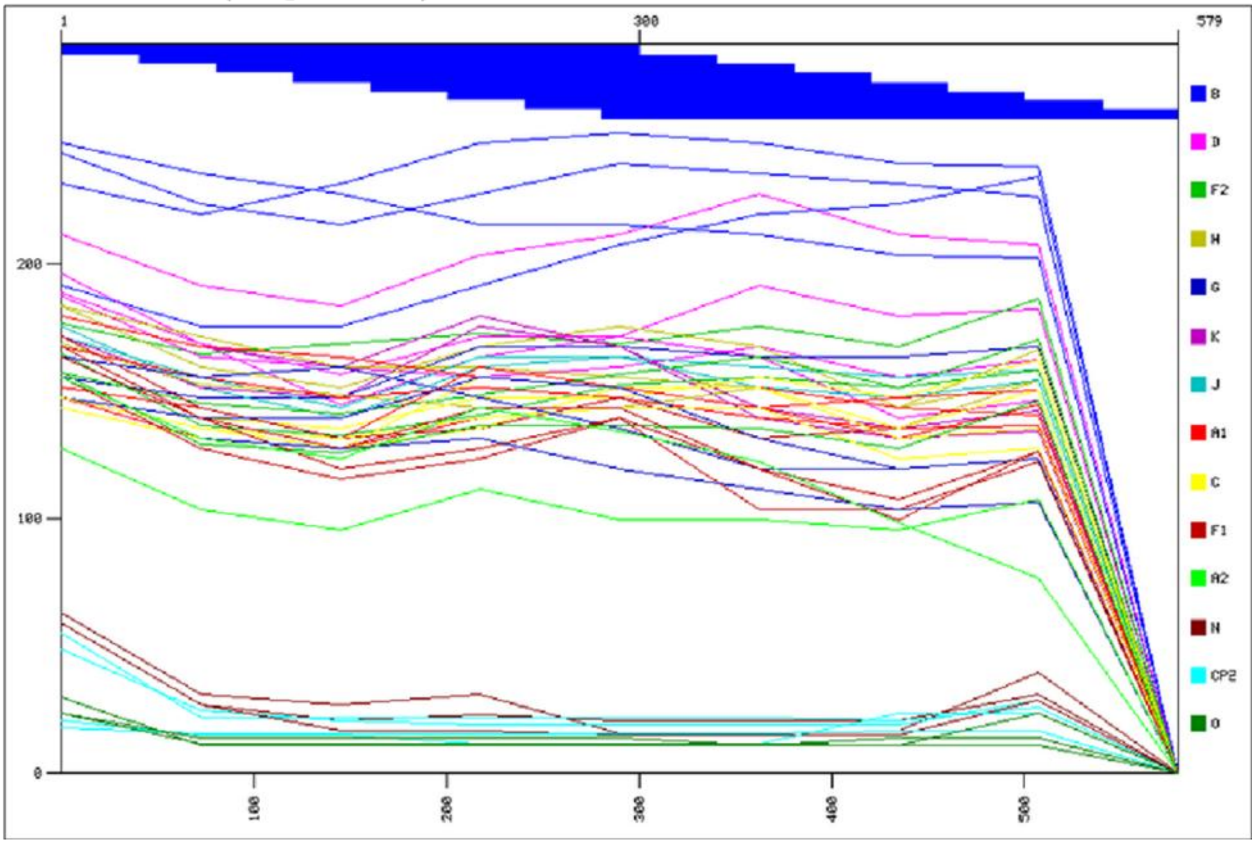

B **VifS1 (EU700276) – Vif C**

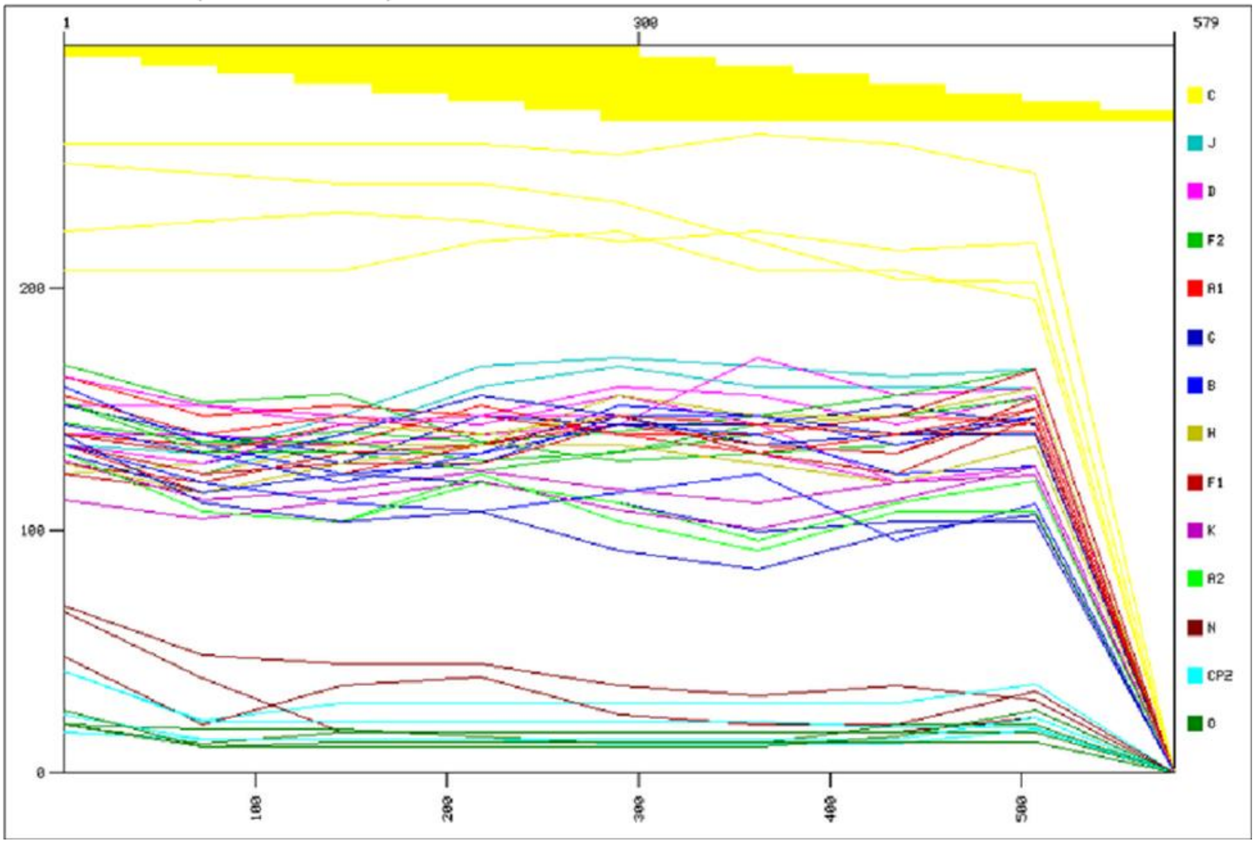

C VifA6 (FJ429356) – Vif B/C

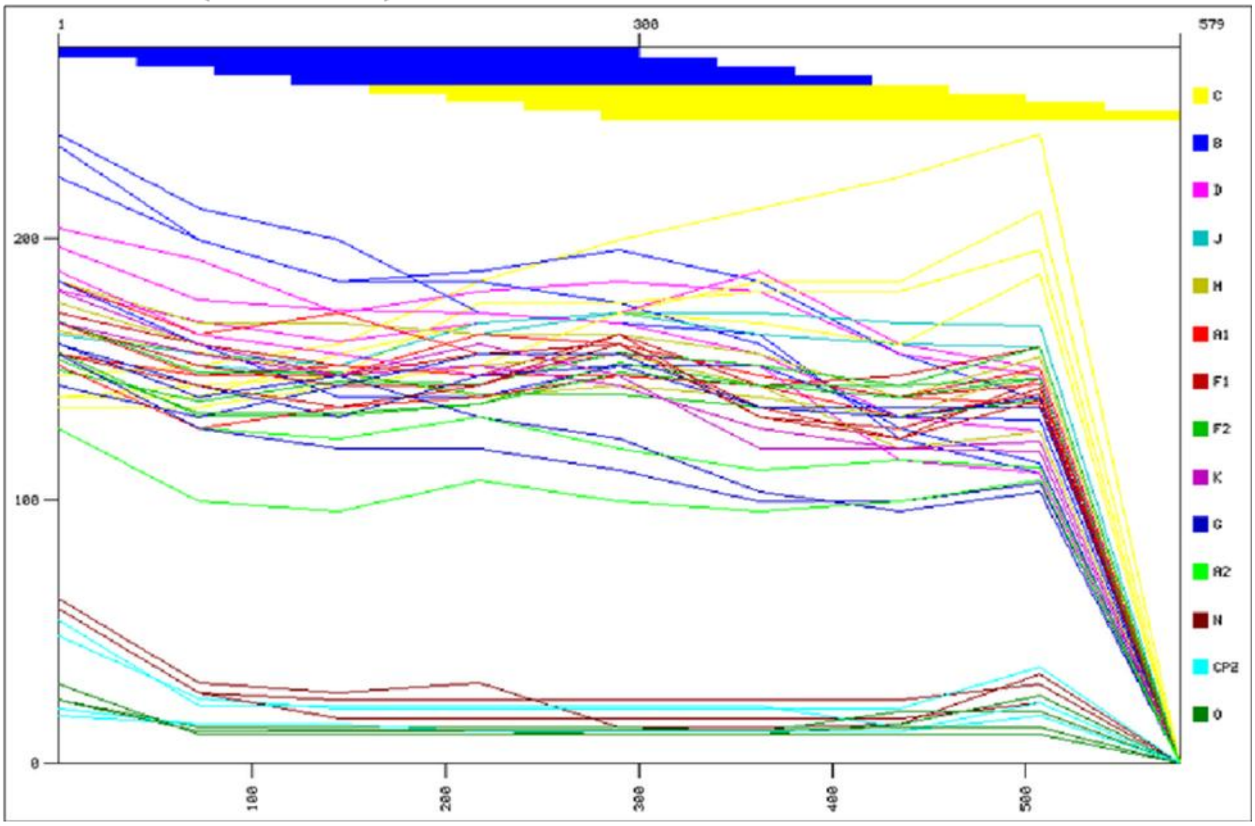

Supplemental Figure S2. **Recombination events in Vif variants.** A) VifD64 is a representative of Vif B variant. B) VifS1 is a representative of Vif C variant. C) VifA6 is a representative of Vif B/C recombinant. The yellow line represents subtype C and the blue line represents subtype B.

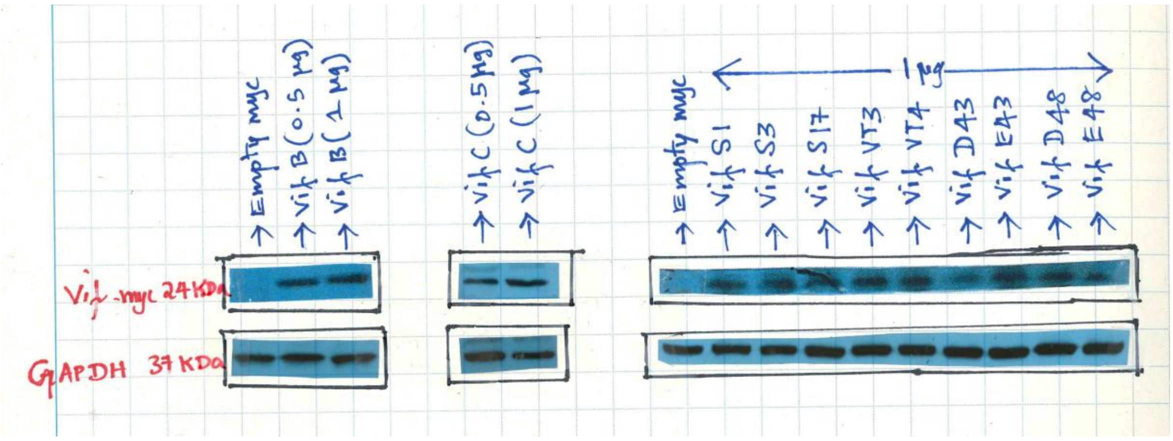

Supplemental Figure S3. **Protein expression of Vif variants.** Unprocessed original Scans of western blots with molecular size (KDa).

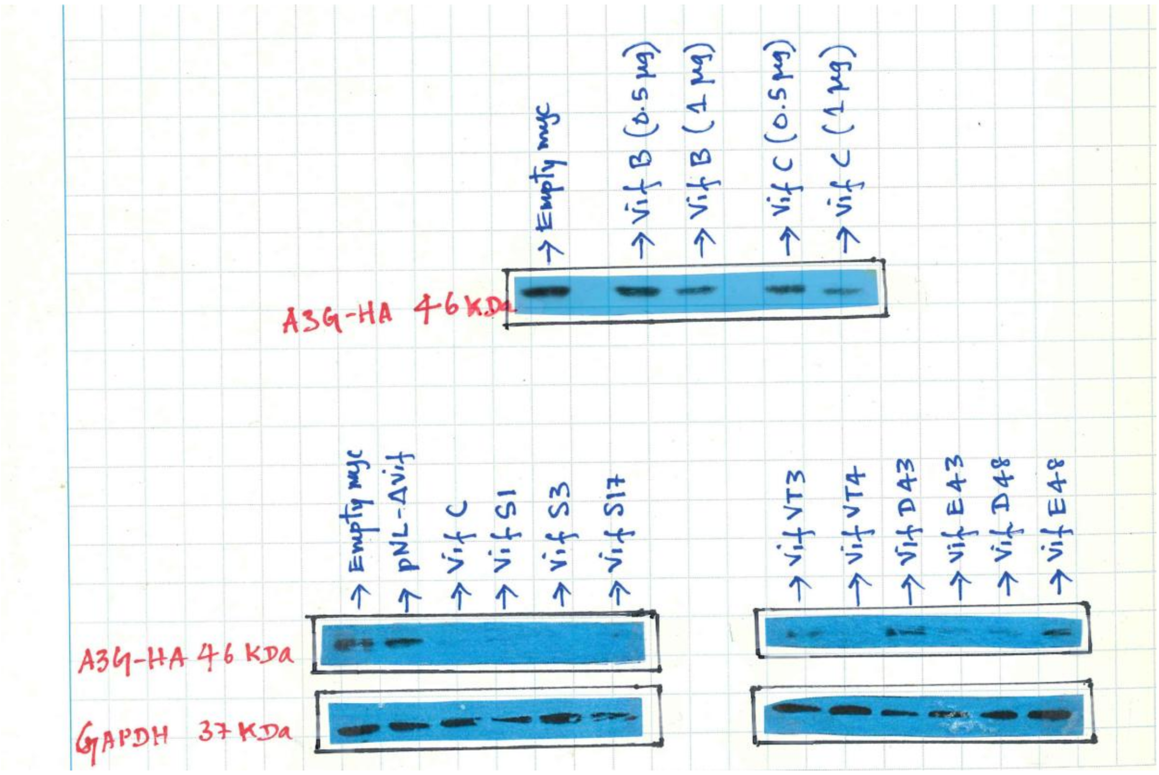

Supplemental Figure S4. **APOBEC3G degradation by Vif variants.** Unprocessed original Scans of western blots with molecular size (KDa).

Samples	Age (yrs)	Sex	Route of transmission	Positive since detection	ART status	CD4 counts	Predicted Subtypes
S1	33	M	Heterosexual	2005	ART –ve	364	C
S2	37	M	Heterosexual	2006	ART –ve	NA	B
S3	35	F	Heterosexual	2004	ART +ve	253	C
S4	23	F	Heterosexual	2007	ART –ve	NA	B/C
S5	29	M	Heterosexual	2008	ART +ve	111	B/C
S6	36	M	Heterosexual	2008	ART –ve	345	B/C
S17	38	M	Heterosexual	2008	ART +ve	234	C
S19	27	F	Heterosexual	2007	ART +ve	320	C
S57	36	M	Heterosexual	2007	ART +ve	354	C
S80	24	F	Heterosexual	2008	ART –ve	481	B
S81	34	M	Heterosexual	2008	ART +ve	211	B
VT1	24	F	Heterosexual	2007	ART +ve	152	B/C
VT2	4	M	Vertical	2007	ART +ve	727	B/C
VT3	30	F	Heterosexual	2008	ART –ve	233	B/C
VT4	8	M	Vertical	2007	ART +ve	804	B/C
VT5	38	F	Heterosexual	2006	ART –ve	96	B/C
VT6	6	M	Vertical	2006	ART –ve	1048	B/C
VTD8	27	F	Heterosexual	2006	ART –ve	475	B
VTE8	5	F	Vertical	2006	ART +ve	870	B
D1	30	F	Heterosexual	2008	ART –ve	419	B
E1	9	M	Vertical	2008	ART +ve	NA	B
D2	30	F	Heterosexual	2008	ART +ve	403	B
E2	6	M	Vertical	2008	ART –ve	972	B
D19	30	F	Heterosexual	2008	ART +ve	310	B
E19	10	M	Vertical	2008	ART +ve	720	B
D43	31	F	Heterosexual	2008	ART +ve	430	B
E43	8	M	Vertical	2008	ART –ve	734	B
D47	28	F	Heterosexual	2008	ART +ve	390	B
E47	7	M	Vertical	2008	ART +ve	732	B
D48	27	F	Heterosexual	2008	ART +ve	310	B
E48	8	M	Vertical	2008	ART +ve	720	B
D63	30	F	Heterosexual	2008	ART –ve	212	B
E63	10	M	Vertical	2008	ART +ve	458	B
D64	35	F	Heterosexual	2008	ART +ve	403	B
E64	7	M	Vertical	2008	ART +ve	572	B
A4	30	M	Heterosexual	2004	ART –ve	351	B
A5	28	F	Heterosexual	2008	ART –ve	972	B
A6	28	F	Heterosexual	2006	ART –ve	1046	B/C
A7	35	M	Heterosexual	2008	ART –ve	519	B
A8	39	M	Heterosexual	2006	ART –ve	447	B
A9	35	M	Heterosexual	2006	ART –ve	440	B
N11	32	M	Heterosexual	2010	ART +ve	140	B
N12	27	F	Heterosexual	2010	ART +ve	226	C
N13	39	F	Heterosexual	2010	ART +ve	104	C
N14	40	M	Heterosexual	2010	ART +ve	198	B
N15	50	M	Heterosexual	2010	ART +ve	74	C
N16	30	M	Heterosexual	2010	ART +ve	256	B
N17	40	M	Heterosexual	2010	ART +ve	114	B
N45	30	M	Heterosexual	2010	ART –ve	153	C

N46	25	M	Heterosexual	2010	ART –ve	125	B
N47	35	F	Heterosexual	2010	ART –ve	339	C
N48	24	F	Heterosexual	2010	ART –ve	600	C
N49	32	M	Heterosexual	2010	ART –ve	717	B
N50	32	F	Heterosexual	2009	ART –ve	203	C
N51	25	M	Heterosexual	2010	ART –ve	NA	B/C
N52	24	F	Heterosexual	2010	ART –ve	165	C
N53	30	M	Heterosexual	2010	ART –ve	64	C
N54	35	F	Heterosexual	2009	ART –ve	NA	B
N55	36	M	Heterosexual	2010	ART –ve	39	B
N56	30	M	Heterosexual	2009	ART –ve	110	C
N57	27	F	Heterosexual	2009	ART –ve	NA	C
N58	45	F	Heterosexual	2009	ART –ve	156	B/C
N59	45	F	Heterosexual	2010	ART –ve	56	B
N60	37	M	Heterosexual	2008	ART –ve	93	B
N61	24	M	Heterosexual	2008	ART –ve	NA	C
N62	35	M	Heterosexual	2010	ART –ve	119	C
N63	30	M	Heterosexual	2010	ART –ve	159	B
N64	42	F	Heterosexual	2010	ART –ve	146	B
N65	32	M	Heterosexual	2010	ART –ve	NA	B/C
N66	36	M	Heterosexual	2009	ART –ve	591	C
N67	35	M	Heterosexual	2010	ART –ve	NA	B
N68	25	F	Homosexual	2009	ART +ve	80	C
N69	25	F	Heterosexual	2007	ART –ve	433	C
N70	22	M	Heterosexual	2008	ART –ve	78	B/C
N71	32	F	Heterosexual	2007	ART +ve	139	C
N72	27	F	Homosexual	2008	ART +ve	131	C
N73	39	M	Heterosexual	2007	ART +ve	218	B
N74	40	M	Heterosexual	2008	ART +ve	198	C
N75	50	M	Heterosexual	2010	ART +ve	74	B
N76	30	M	Heterosexual	2010	ART –ve	304	C
N77	40	M	Heterosexual	2010	ART –ve	63	C
N78	30	F	Heterosexual	2010	ART –ve	358	B
N79	25	F	Heterosexual	2007	ART +ve	55	C
N80	34	M	Heterosexual	2009	ART +ve	83	B/C
N81	21	F	Heterosexual	2009	ART +ve	176	C
N82	32	F	Heterosexual	2008	ART –ve	774	C
N83	50	F	Heterosexual	2009	ART –ve	225	B
N84	40	F	Heterosexual	2007	ART +ve	265	B
N85	51	M	Heterosexual	2007	ART +ve	85	C
N86	32	M	Heterosexual	2007	ART +ve	185	B
N87	41	F	Heterosexual	2007	ART +ve	114	B
N88	48	M	Heterosexual	2008	ART –ve	451	C
N89	13	M	Vertical	2008	ART –ve	422	B
N90	32	M	Heterosexual	2009	ART +ve	111	C
N91	04	M	Heterosexual	2009	ART –ve	301	B
N92	48	M	Heterosexual	2008	ART +ve	103	C
N93	19	F	Heterosexual	2008	ART –ve	116	B
N94	06	F	Vertical	2009	ART –ve	227	C
N95	08	M	Vertical	2009	ART –ve	547	C
N96	38	M	Heterosexual	2009	ART +ve	90	B/C
N97	35	F	Heterosexual	2009	ART –ve	442	B

N98	03	F	Vertical	2010	ART –ve	429	B
N99	25	M	Heterosexual	2009	ART –ve	317	C
N100	29	F	Heterosexual	2005	ART +ve	114	B
N101	48	M	Heterosexual	2005	ART +ve	252	C
NA (Not Available); ART –ve (naive); ART +ve (positive); Letter D denotes mother and E denotes child							

Supplemental Table S1. Clinical data for HIV-1 infected patients from North India (n=105).

Mutations	Frequency
**G18T**	0.111
G19T	0.027
**A25G**	0.111
A43C	0.027
G51A	0.027
G56A	0.722
**A57C**	0.722
**G63C**	0.166
A66G	0.027
**T69A**	0.666
A91G	0.277
G110A	0.666
**A111C**	0.722
A119G	0.027
**A121G**	0.166
A125T	0.027
T126C	0.027
**A135C**	0.277
C138T	0.722
A139C	0.027
**C142A**	0.722
T144C	0.027
A148G	0.027

A149G	0.027
A151T	0.027
G155T	0.027
G160A	0.027
G163T	0.027
A165C	0.027
A167C	0.027
A169T	0.027
G181A	0.027
**T183G**	0.333
**G188A**	0.722
**A189T**	0.111
T213G	0.277
A229G	0.027
**G284A**	0.277
**A286G**	0.111
**G292A**	0.444
**C297A**	0.444
A318G	0.277
T330C	0.555
C333T	1.000
**T342G**	0.111
A348G	0.027
G349T	0.027
T354C	0.166
G362T	0.027
A363G	0.444
**G367A**	0.555
**A380G**	0.555
G385T	0.027
**C391T**	0.111

A401G	0.027
A419G	0.027
A440G	0.027
G450A	0.027
T457C	0.444
**A472C**	0.555
A473G	0.027
A479G	0.027
**G496A**	0.444
**G500A**	0.444
G513A	0.444
T516C	0.500
**G528T**	0.333
**C539T**	0.333

Supplemental Table S2. Nucleotide changes in Vif variants from North India

Samples	dN/dS (Consensus C)	dN/dS (Consensus B)	Predicted Subtypes	Selection
VifS1	0.3194	0.3898	C	Purification
VifS3	0.3380	0.4310	C	Purification
VifS17	0.3240	0.4120	C	Purification
VifD47	0.6169	0.3455	B	Purification
VifE47	0.5830	0.3323	B	Purification
VifD48	0.5605	0.3007	B	Purification
VifE48	0.5769	0.3120	B	Purification
VifA6	0.3384	0.3474	B/C	Purification
VifVT4	0.3415	0.3530	B/C	Purification
VifVT3	0.3304	0.3341	B/C	Purification

Supplemental Table S3. dN/dS calculation for unique Vif variants
